# Oceanic and super-deep continental diamonds share a transition zone origin and mantle plume transportation

**DOI:** 10.1038/s41598-021-96286-8

**Published:** 2021-08-20

**Authors:** Luc S. Doucet, Zheng-Xiang Li, Hamed Gamal El Dien

**Affiliations:** Earth Dynamics Research Group, TIGeR, School of Earth and Planetary Sciences, Curtin University, Perth, WA 6845 Australia

**Keywords:** Geochemistry, Geodynamics, Geology, Mineralogy, Petrology, Tectonics

## Abstract

Rare oceanic diamonds are believed to have a mantle transition zone origin like super-deep continental diamonds. However, oceanic diamonds have a homogeneous and organic-like light carbon isotope signature (δ^13^C − 28 to − 20‰) instead of the extremely variable organic to lithospheric mantle signature of super-deep continental diamonds (δ^13^C − 25‰ to + 3.5‰). Here, we show that with rare exceptions, oceanic diamonds and the isotopically lighter cores of super-deep continental diamonds share a common organic δ^13^C composition reflecting carbon brought down to the transition zone by subduction, whereas the rims of such super-deep continental diamonds have the same δ^13^C as peridotitic diamonds from the lithospheric mantle. Like lithospheric continental diamonds, almost all the known occurrences of oceanic diamonds are linked to plume-induced large igneous provinces or ocean islands, suggesting a common connection to mantle plumes. We argue that mantle plumes bring the transition zone diamonds to shallower levels, where only those emplaced at the base of the continental lithosphere might grow rims with lithospheric mantle carbon isotope signatures.

## Introduction

The vast majority of diamonds have grown in the old continental lithospheric mantle between 150 and 300 km depths^1^ and are found in mantle xenoliths in kimberlites, lamproite, lamprophyres and related placer deposits. The positions of these deposits relative to plume magmatism and the large low sheared wave velocity province (LLSVP)^2^ suggest that they are related to mantle plume events^2–5^. A small fraction (1%) of the continental diamonds have mineral inclusions that suggest a deeper origin of between ∼ 300 and 1000 km depths^6^, and are known as super-deep or sub-lithospheric continental diamonds^7^. Super-deep continental diamonds have extremely variable carbon isotopic compositions (δ^13^C from − 25 to + 3.5‰)^8–11^, in contrast to a dominant mode of carbon isotope at around − 5‰ exhibited by lithospheric peridotitic and eclogitic diamonds^12^.

**Figure 1:**
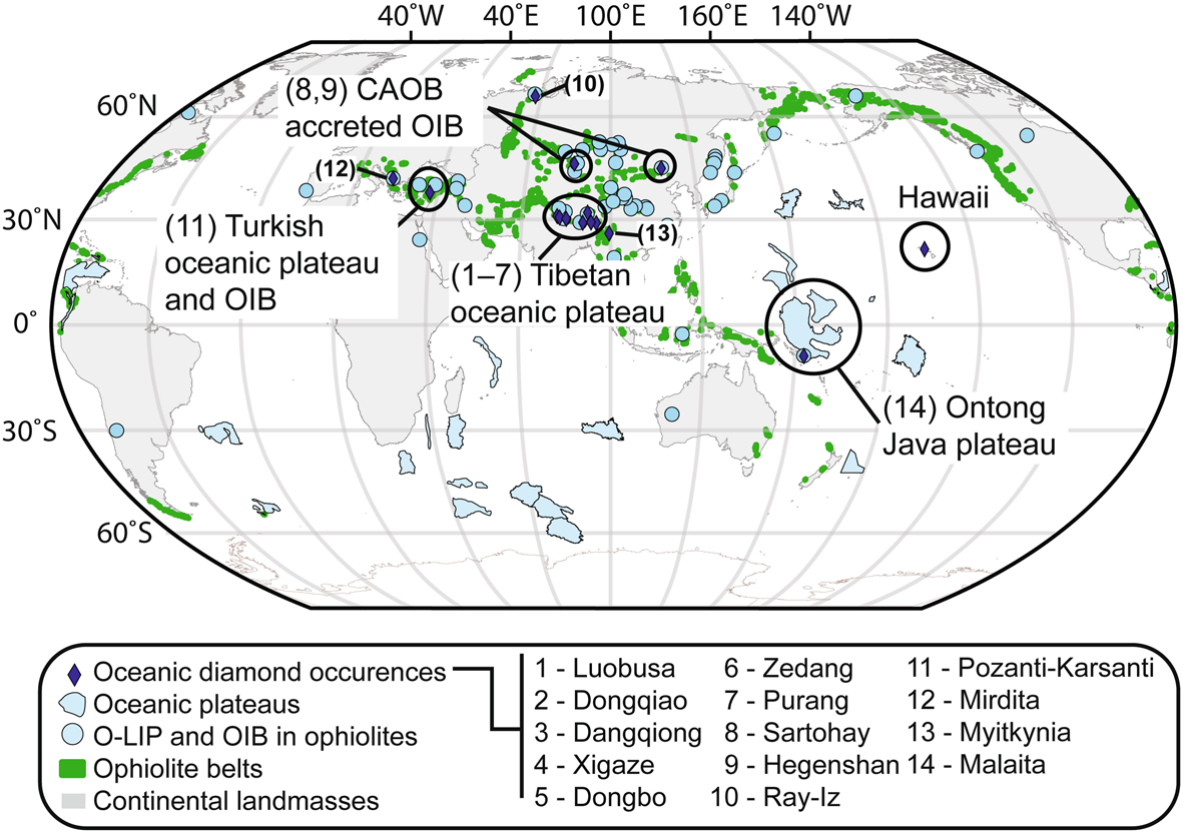
Map showing oceanic diamond and oceanic mantle plume occurrences. The data are from Lian and Yang^16^ and Doucet et al.^33^. The world map is made using GPlate 2.2 open-source software (licensed under the GNU General Public License version 2 https://www.gplates.org/) with open-source coastline data from Matthews et al.^34^ (licensed under Creative Common Attribution 4.0 International License https://www.earthbyte.org/category/resources/).

An even smaller fraction (<< 1%) of diamonds are found in the oceanic lithosphere sampled by mantle xenoliths or preserved in ophiolitic belts worldwide^13–17^ (Fig. 1). Such oceanic diamonds share morphological similarities with synthetic diamonds, e.g., they are euhedral to subhedral with cubo-octahedral shape and low nitrogen aggregation, which led some to question their natural origin^18,19^. However, oceanic diamonds display features, such as inclusions (e.g., coesite and feldspar), presence of moissanite (SiC), and a large continuous range of δ^15^N isotopic composition (− 5.6‰ to + 28.7‰), not found in synthetic diamonds^20–22^. In situ occurrence of such natural diamonds have also been demonstrated by thin-section in situ petrographic observations^23,24^. Some studies proposed that diamonds found in ophiolites could have resulted from serpentinisation processes^22,25^. However, the presence of ultra-high-pressure and highly reduced mineral phases in such diamonds suggests their formation at > 300 km depths^26^, which cannot be explained by serpentinization^27^. Moreover, although diamond nano-particles can form under thermodynamic instability and low pressure–temperature conditions^28,29^, this mechanism is incompatible with the nano- to micro-meter sizes of the oceanic diamonds^30^, and cannot reproduce fluid inclusions with complex compositions (in Na, Cl, K) similar to kimberlitic and ultra-high pressure (UHP) metamorphic diamonds^31,32^.

On the other hand, these oceanic diamonds share similar characteristics to super-deep continental diamonds in that they, in general, are microdiamonds (< 1 mm in size^35^, except for the exceptionally large CLIPPIR diamonds^36^) and are associated with a range of ultra-high-pressure and highly reduced mineral phases (e.g., coesite, kyanite, UHP nitride, SiC, Ni–Mn alloys, Fe–Si and Fe–C)^12,15,16^. It is thus believed that oceanic diamonds and super-deep continental diamonds formed in the volatile-rich regions of the mantle, most likely in the transition zone (410–660 km)^12,16^. It is widely accepted that such diamonds (and related ultra-high pressure and reduced mineral phases) formed from an oxidized, CO_3_-rich melt^37–41^ or reduced fluids produced when subducted slabs melted after entering the transition zone, where the pressure is 15–16 GPa, temperature ~ 1600 °C, and volatile contents 1–1.5 wt%^36,42,43^.

**Figure 2:**
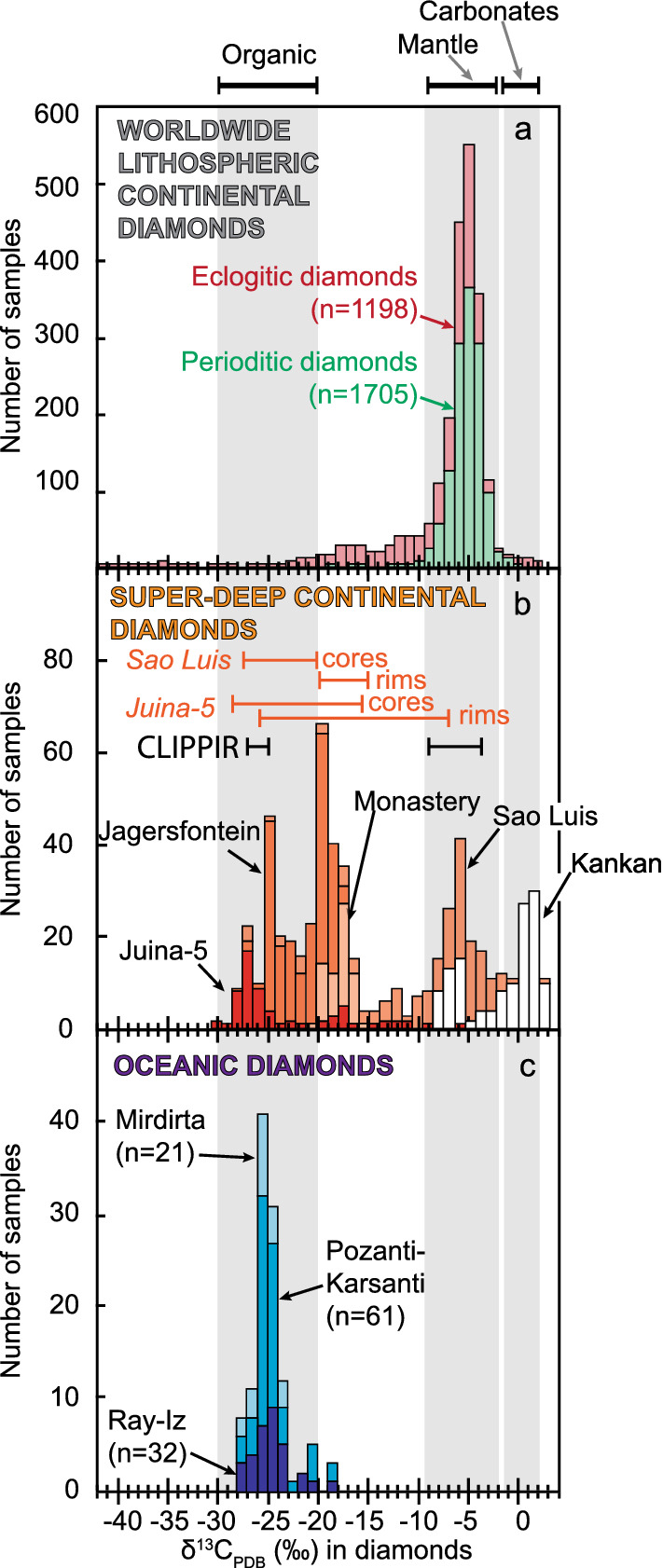
Carbon isotope composition of diamonds (expressed as δ^13^C relative to Pee Dee Belemnite). (**a**) Worldwide lithospheric continental diamonds^12^ showing eclogitic (pink) and peridotitic diamonds (green). (**b**) Super-deep continental diamonds^8–11^ from Juina-5, Jagersfontein, Monastery, Sao Luis and Kankan. Also shown are the carbon isotope ranges for the cores and rims of the Sao-Luis^8^ and Juina-5^10^ diamonds, and data reported for seven fragments of CLIPPIR diamonds^36^. (**c**) Oceanic diamonds from Mirdirta^32^, Pozanti-Karsanti^44^ and Ray-Iz^30^. The carbon isotope range for the mantle is from Deines^48^, the ranges for carbonates and organic matter are from studies of sedimentary rocks of the entire geological record^49–51^.

Despite their similarities, oceanic diamonds and super-deep continental diamonds do display significant differences. First, oceanic diamonds found in ophiolites occur as inclusions in podiform chromitites, which is different from the occurrence of superdeep continental diamonds. Second, oceanic diamonds show a homogeneous carbon isotopic composition (δ^13^C from − 28 to − 20‰)^30,32,44^ (Fig. 2c) whereas super-deep continental diamonds exhibit extreme isotopic variabilities (δ^13^C from − 25 to + 3.5‰)^8–11^ (Fig. 2b). Third, some superdeep diamonds from Juina have oxidized inclusions such as carbonate minerals (magnesite, eitelite, nyerereite and nahcolite)^45–47^, which are not found in oceanic diamonds. Hence, the relationship between super-deep continental diamonds and oceanic diamonds remains elusive, and it is unclear how the oceanic diamonds get incorporated into the oceanic upper mantle before being brought to the surface either as part of mantle xenoliths or part of ophiolites.

Two main mechanisms have been proposed to explain the transportation of oceanic diamonds from the transition zone to the upper mantle: (1) subduction return flow^52,53^, or (2) mantle upwelling^15^. In the first model, gravity causes the subducting slab to roll back, which triggers return flows that brings the diamonds and ultra-high-pressure minerals to the shallower levels within the subduction channel. In the second model, mantle upwellings, possibly induced by the pondering of plume heads on the transition zone, brings the diamonds and ultra-high-pressure minerals to the lithospheric mantle, fragments of which are later accreted onto the continental margin during subduction.

In this contribution, we aim to achieve a coherent mechanism for the origin of both the oceanic diamonds and the super-deep continental diamonds, including processes that brought them to the surface. We test the various models by investigating the oceanic diamond record together with a compilation of oceanic large igneous provinces (O-LIPs) and ocean island basalts (OIBs) database, or O-LIPdb^33^, which includes geological and geochemical evidence for plume-related materials in ophiolites^54^. We show that most oceanic diamonds found in ophiolite belts are not associated with classic ophiolitic sequences that represent the normal oceanic lithosphere formed at mid-ocean ridges; instead, they are associated with plume-modified O-LIP or OIB lithospheric fragments preserved in ophiolite belts.

## Results

We observe a striking correlation between the occurrence of oceanic diamonds in either present-day oceanic rocks^13,14^ or ancient ophiolites^15,16^, and the oceanic mantle plume record^33^ (Fig. 1; Table 1). The correlation is most obvious when only considering the modern oceanic diamonds, as both the Hawaiian and Malaita islands examples (Fig. 1) are known to be of plume origin. Diamond inclusions are found in garnet-bearing xenoliths from the Malaita islands, the exhumed portion of the southwest Ontong Java oceanic plateau. Both seismic data and mantle xenolith studies there point to a mantle lithosphere thicker than 130–140 km^55–58^. Such a thickened oceanic lithosphere, together with the highly depleted nature of the mantle lithosphere in these two regions, is consistent with their plume origin^59^.

**Table 1:** Oceanic diamond occurrences and relationship with oceanic large igneous provinces and ocean islands.

	Lat. decimal	Long. decimal	Magmatic age m.y	Emplacement age m.y	Location	Diamond discovery	Plume type	Tectonic setting reference	Location name	Tectonic settings of ophilolite fragments	Note
*Diamonds in xenoliths*											
Salt-lake crater	21.47	− 158.00	0.44		O'ahy, Hawaii	Wirth and Rocholl ^14^	OIB	Wirth and Rocholl^14^	Hawaii		
Malaita	− 9.05	161.19	34		Malaita Island	Collerson et al.^13^	O-LIP	Collerson et al.^13^	Ontong Java		
*Diamond-bering ophiolites*											
Xigaze	29.16	88.88	125	100–65	Yarlung Zhangbo belt, Tibet, China	Xiong et al. (2016)	O-LIP	Yang and Dilek^61^, Zhang et al.^60^	Tibetan oceanic plateau	(5)(7)	Dismembered ophiolite, made of several bodies along the Yarlung Zhangbo belt
Purang	30.66	80.95	125	100–65						(5)(7)	
Dangqiong	30.23	83.19	125	100–65						(5)(7)	
Lubuosa	29.04	93.39	130	100–65						(5)(7)	
Dongbo	31.05	80.17	130	100–65						(5)(7)	
Zedang	29.18	91.61	160	100–65						(5)(7)	
Dongqiao	32.00	90.44	196	100–65						(5)(7)	
Hegenshan	44.75	116.39	295	244	Inner-Mongolia, China	Huang et al. (2015)	OIB	Yang et al. (2015a)	Hegenshan	(5)(7)	Dismembered ophiolite
Sartohay	46.08	84.97	430	316	West Jungar, China	Tian et al. (2015)	OIB	Miao et al.^63^	West Jungar	(1)(3)(5)(6)(7)	Dismembered ophiolite
Mirdita	41.83	21.00	160	100	Albania	Xiong et al. (2017)	OIB	Gaggero et al.^65^	Mirdita	(1)(3)(6)(7)(8)	Semi-complete section
Myitkyina	26.00	97.83	170	100–65	Myanmar	Chen et al.^66^	n.a		n.a	(5?)(6?)(7)	Dismembered ophiolite
Rai-Iz	66.79	64.99	420	400	Polar-Ural	Yang et al. (2015)	n.a		n.a	(3)(6?)(7)	Dismembered ophiolite
Pozanti-Karsanti	37.70	35.36	95	100–65	Turkey	Lian et al. (2017)	n.a		Eastern Tauride belt	(4)(6)(7)(8)	Semi-complete section

*m.y.* million years, *O-LIP* oceanic large igneous provinces, *OIB* ocean island basalts, *Lat.* latitude, *Long*. longitude.

(1), continental arc; (3), older oceanic arc; (4), oceanic arc; (5), oceanic plateau; (6), seamount and ocean island; (7), oceanic crusr; (8), mid-ocean ridge.

**Figure 3:**
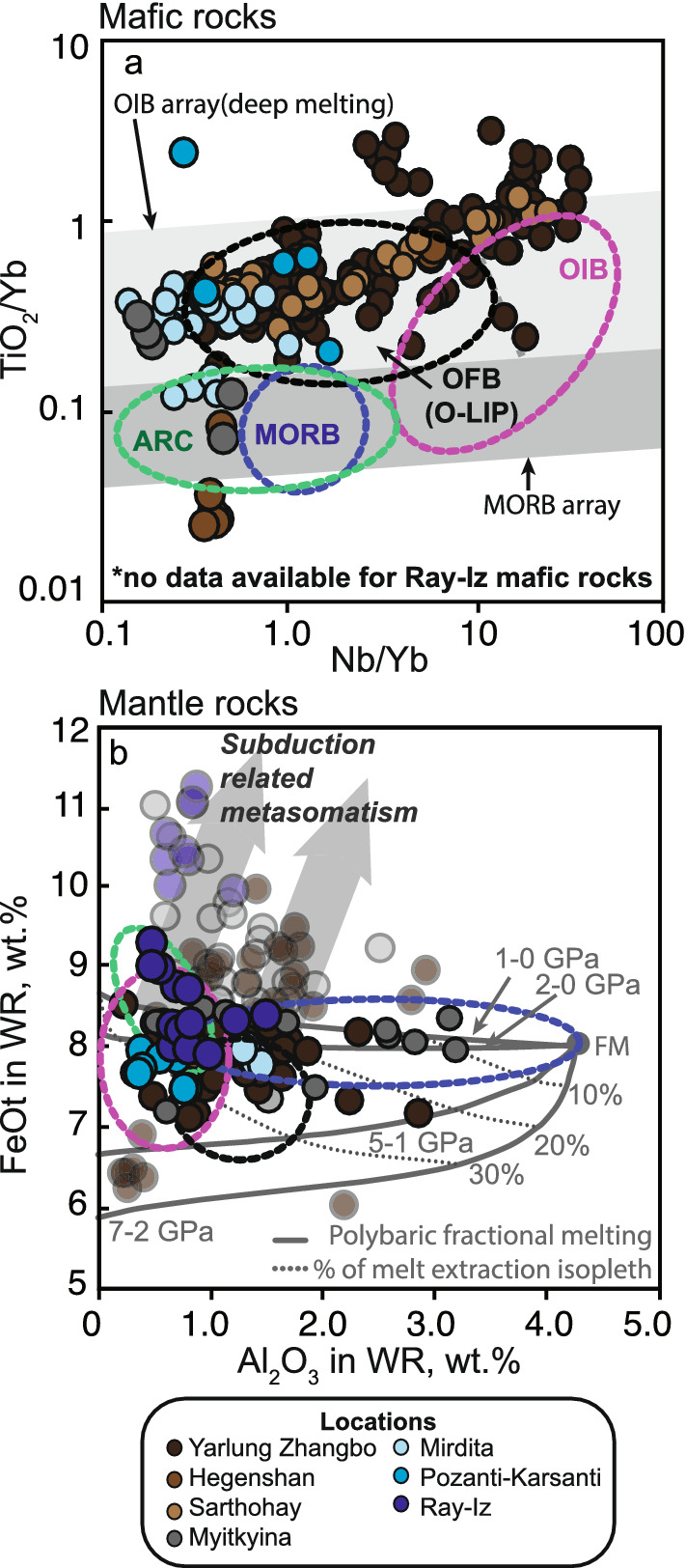
Geochemical characteristics of mafic and ultramafic rocks from diamond-bearing ophiolites plotted against fields of tectonic settings. (**a**) Nb/Yb versus TiO_2_/Yb in mafic rocks, (**b**) Al_2_O_3_ versus FeOt (in wt. %) in mantle rocks. See Supplementary Data Figure S2 for definition and sources of the tectonic setting discrimination fields. Also shown in (**a**) are the oceanic basalt (MORB) and ocean island basalt (OIB) discriminant fields (arrays) of Pearce^67^, and in (**b**) the experimental melting residue for polybaric fractional melting of fertile mantle^68^, with faded color shades showing data affected by subduction-related metasomatism^69^. Dotted ovals show fields of tectonic settings following the same color code as in (**a**).

From the ophiolite record (Table 1; Fig. 1), the Tibetan ophiolites from the Yarlung-Zhanbo belt, which are the remnants of the lithospheric mantle of Tethyan oceanic plateau(s)^60,61^ (Fig. 3), provide the most frequent occurrences of oceanic diamonds (Fig. 1). The Sartohay (part of the Darbut ophiolitic melange) and Hegenshan ophiolites are associated with accreted OIBs in the Central Asian Orogenic Belt (CAOB) in the West Jungaar suture zone^62^ and the Inner Mongolia-Daxinganling orogenic belt^63^, respectively, both exhibiting O-LIP characteristics^33^. The Pozanti-Karsanti (also known as Aladag) ophiolite in Turkey is part of the eastern Tauride belt, and is characterised as an accreted OIB or oceanic plateau^64^ similar to the Mirdita ophiolite nappe^65^. Although the original authors interpreted the Ray-Iz and Myitkynia ophiolites to be of suprasubduction origin without plume involvement, we found that the mafic and ultramafic rocks from these two ophiolites share similar geochemical features to the plume-modified oceanic lithosphere (Fig. 3 and Figures S3 and S4). Diamonds from these two ophiolites also share similar features as the O-LIP/OIB-related diamonds: the presence of ultra-high pressure minerals and highly-reduced phases, and very low δ^13^C (i.e. − 30 to − 20‰ for the Ray-Iz diamonds) (Fig. 2)^27,66^. We thus consider them to be plume-related diamond-bearing ophiolites as well.

**Figure 4:**
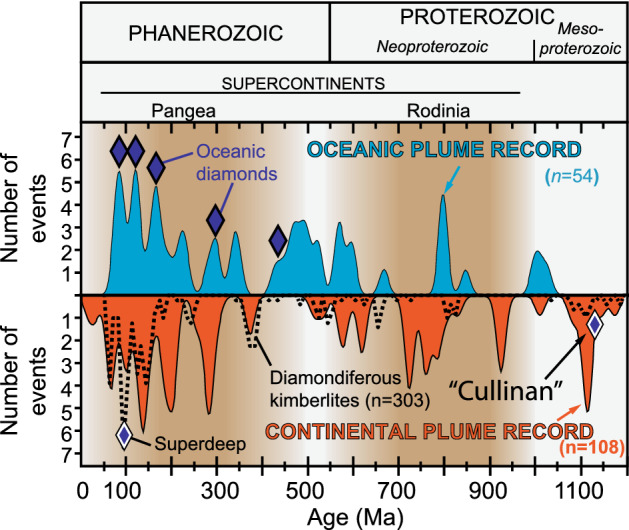
Distribution of diamond occurrences through time. The occurrences of oceanic diamonds (dark blue diamonds)^13,14,16^ and super deep diamonds (blue diamonds with white rims)^72,73^ are plotted against the time distributions of diamondiferous kimberlites (dotted curve)^73^, and oceanic (light blue) and continental (orange) mantle plume occurrences for the last 1200 million years^33^ and the life cycles of supercontinents Pangea and Rodinia^74^. Note that the majority of the known superdeep diamonds were brought up to the surface by 110–90 Ma kimberlites. The “Cullinan” (CLIPPIR) diamonds are from the much older Premier kimberlite (1153 Ma)^75^.

In addition, the magmatic ages of diamond-bearing ophiolites (Table 1) also coincide with the peaks of oceanic mantle plume activities for the last 500 million years at 430, 395, 165, 125 and 95 million years ago (Fig. 4)^33^. These peaks reflect an increase in mantle plume activity with time, interpreted to be the result of global mantle dynamics driven by the supercontinent cycle^70,71^. This observation further reinforces a plume connection for oceanic diamonds. A similar general correlation between continental plume record and diamondiferous kimberlites suggest the same connection for continental diamonds^72,73^.

## Discussion

Super-deep continental diamonds and oceanic diamonds share some characteristics. However, the extremely variable carbon isotopic composition of super-deep continental diamonds (δ^13^C ranges from − 28 to 3‰, average of − 8 ± 9‰)^8,10,12^ from one locality to another (Fig. 2b) is in stark contrast to the relatively homogenous carbon isotopic composition of oceanic diamonds (δ^13^C range from − 28 to − 19‰, average of − 25 ± 4‰; Fig. 2c), leading previous researchers to believe that they are of distinct carbon sources and therefore geneses and origins. A detailed examination reveals that some individual diamonds from the Juina-5^10^ and Sao Luis (Brazil)^8^ continental super-deep diamonds exhibit carbon isotopic zonation, featuring very light C isotope fractions in their cores (δ^13^C from − 28 to − 20‰) and heavier, mantle-like C isotope compositions in the rims (δ^13^C − 15‰ to − 5‰)^8,10^ (Fig. 2b). The Kankan diamonds represent a rare exception with dominantly >− 5‰ C isotope (Fig. 2b). This general trend of very light isotope in the cores and heavy isotope in the rims is generally coupled with distinct cathodoluminescence colours between the cores and rims, interpreted as indicating pulses of diamond growth^10^.

Previously proposed models to explain the variability of carbon isotopic composition in super-deep continental diamonds include (1) primordial isotopic variability inherited from Earth's accretion^76^, (2) distinct carbon sources for the cores and rims (organic and inorganic)^49–51^, and (3) isotopic fractionation of carbon in the mantle^77,78^.

The fact that (1) the highly negative carbon isotope values for the cores of the super-deep continental diamonds from Juina-5 and Sao Luis (δ^13^C − 24 ± 6‰), and the overall homogeneous negative values for the superdeep diamonds from Jagersfontein (δ^13^C up − 20 ± 4‰)^11^ and Monastery (δ^13^C up − 17 ± 1‰)^11^, are comparable to those of the relatively homogeneous carbon isotopic composition of oceanic diamonds (δ^13^C − 25 ± 4‰) (Fig. 2), and (2) they are believed to be of an organic origin from subducted slabs, can rule out the possibility of primordial origin and variability. The model involving fractionation processes during degassing of CO_2_ (enriched in ^13^C) and nitrogen is not supported either due to the lack of correlation between δ^13^C and nitrogen in various growth zones^8^. This leads us to hypothesize that the very light cores of Juina-5 and Sao Luis super-deep diamonds, and superdeep diamonds from Jagersfontein and Monastery, share a common origin with the oceanic diamonds, as reflected by their carbon isotope signatures (Fig. 2b,c). Furthermore, the organic matter-like (δ^13^C between − 30 and − 20‰^49,50^) very light isotopic composition of such diamonds (Fig. 2b,c) from the mantle transition zone argues for a common organic carbon origin. Most of the carbon (90%) in the oceanic lithosphere is stored in the altered crust, while organic matter represents only a small fraction (< 10%) of the available carbon^79,80^. In view of the distinct organic carbon signature exhibited by the transition zone diamonds, it appears that organic carbon might be the dominant carbon available in the transition zone, and the transition zone likely plays a critical role in carbon cycles^81^.

**Figure 5:**
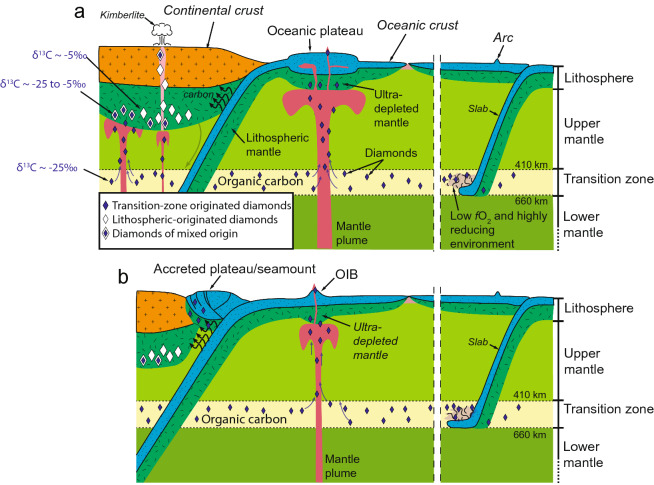
Model for the genesis of three types of diamonds. (**A**) Oceanic and super-deep continental diamonds (cores only) form in the mantle transition zone using subducted organic carbon, and are then brought to the lithospheric levels by mantle plumes. Continental diamonds grow or overgrow (as rims over the super-deep diamonds) in the continental lithosphere. (**B**) Oceanic diamond-bearing rocks are accreted onto continental margins as fragments of obducted oceanic plateaus or ocean islands.

Some mantle plumes are rooted from the lower mantle, whereas others could have a root near the transition zone, possibly as secondary plumes^82,83^, all above the large low-shear-velocity provinces (LLSVPs) in the lower mantle^82,84,85^. We envisage that mantle upwellings, caused by plumes^86,87^, entrain microdiamonds formed in the transition zone and transport them (and other associated ultra-high pressure minerals) to shallower levels^88–93^. In contrast, mantle convection around normal mid-ocean ridges, away from mantle plumes, do not contain such diamonds and ultra high-pressure minerals (Fig. 5). The high degree of melt extraction induced by mantle plumes is responsible for the formation of thicker (100–140 km)^55,56^ and highly depleted (in iron and other incompatible elements) oceanic lithospheric mantle^59^. Buoyancy caused by such depletion, along with plume-induced thermal buoyancy and the abnormal thickness of such plume-modified oceanic lithosphere^94^, makes it more resistant to subduction^95^, leading to components of it being accreted onto arcs and preserved in orogenic belts^96^. Subduction fluids modify the original chemical signature of the lithospheric mantle by melt-rock interactions at shallow depth during both accretion/obduction and exhumation in the spinel stability field (< 80 km), leading to the formation of large podiform chromite bodies, typical of subduction zones^16^. During the accumulation processes of podiform chromite, oceanic diamonds are incorporated in newly formed high-Cr chromite^97^. Such a process is not only consistent with the deep and highly reduced origin of oceanic diamonds and associated ultra-high-pressure minerals and chromite, but also the subduction origin of the high-Cr podiform chromite.

Our model (Fig. 5) also provides a possible explanation for the extremely variable carbon isotopic composition of the super-deep continental diamonds (Fig. 2b) and the contrasting carbon isotope signatures of the three types of diamonds (Fig. 2). According to our model, mantle plumes bring the same super-deep microdiamonds, with homogeneous carbon isotopic composition, from the transition zone to the lithospheric levels of both the continental and oceanic realms (Fig. 5a) where they can potentially grow. Diamond growth is governed by conditions including the quantity of carbon available (CO, CO_2_, CH_4_), the pressure–temperature condition of the ambient environment (P > 130–150 km for T > 1000 °C), the redox condition (Δlog*f*O_2_ (oxygen fugacity) <−2) which controls the speciation of carbon and its precipitation^98^, and the time available for the growth to occur. The P–T–*f*O_2_ of the lithospheric mantle beneath the oldest continents (aka cratons) is known to favor the growth of diamond^99^. Such lithosphere is typically thicker (up to 300 km), colder (< 900–1000 °C) and reduced (down to Δlog*f*O_2_ <−4)^100^, contains sufficient amounts of carbon^101^, and is able to survive for a sufficiently long time. In such an environment, “purely” continental diamonds (white diamonds in Fig. 5) can grow to gemstone sizes with a homogeneous and predominantly lithospheric carbon isotope signature (δ^13^C − 5‰) (Fig. 2a). Super-deep continental diamonds carried up by plumes (Fig. 5) can also grow rims there that share the same lithospheric carbon isotope signature (δ^13^C − 10 to 0‰), yet their cores, of super-deep origin, retain their original lighter carbon isotopic signature which is the same as that of the oceanic diamonds (δ^13^C − 25 to − 20‰) (Fig. 2).

The P–T–*f*O_2_ and duration of the oceanic plateau and ocean island lithosphere, on the other hand, are rather different although some parameters are still poorly constrained (e.g., a total lack of data on the redox state). The lithospheric mantle of oceanic plateaus and ocean islands is believed to be thinner (< 140 km)^55,58,102^, hotter (1000–1200 °C)^59^, more oxidised (Δlog*f*O_2_ from − 3 to − 1 according to limited data on mid-ocean ridge peridotites)^103^, with less carbon available^79^, and recycled quickly through Wilson cycles. Oceanic microdiamonds, once incorporated in the thickened oceanic lithosphere by plumes, are thus suppressed from growth, or even totally frozen in size, shape (cubo-octahedral) and low aggregation state (Type Ib), due to such unfavorable conditions (Fig. 5)^30^. They therefore still retain their original narrow range and homogeneous carbon isotopic composition (Fig. 2c).

Our model thus provides a processes for the formation and emplacement of three major types of diamonds. Our model differs from that of Yang’s group^17^ in that their model envisages widespread oceanic diamonds in the upper mantle, including in mid-ocean ridge environments, whereas in our model the occurrence of all three major types of diamonds are restricted to rocks linked to mantle plumes as our observations demonstrate (Fig. 1; Table 1). If correct, future work can use oceanic diamonds as a tracer for past oceanic mantle plume records in ophiolites formed through Earth’s history, and to test competing geodynamic models^33,70^. Further testing of our model requires an improved mantle oxidation dataset from oceanic plateaus and ocean islands, and more stable isotope ratio and age data for oceanic diamonds.

## Methods

### Data compilation

We compiled the known occurrences of both present-day oceanic diamonds and those found in ophiolite belts (Table 1)^13,14,16^. The location of each occurrence is presented in Fig. 1. Diamond-bearing mantle xenoliths in present-day oceans provide the most recent (34–0.44 million years old, m.y.) occurrence of oceanic diamonds (Table 1). These modern oceanic diamonds are found in garnet-bearing xenoliths in the Salt Lake crater (0.44 m.y.) near Honolulu, Hawaii^14^, and from the alnoite pipe in the Malaita Islands (34 m.y., Solomon Islands)^13^ (Fig. 1). Diamond-bearing ophiolites contain much older oceanic diamonds (420–95 m.y.) (Table 1)^16^ and include the Pozanti-Karsanti (also known as Aladag) ophiolite (ca. 95 m.y) in the eastern Tauride belt, Turkey; the Tibetan ophiolites (ca. 196–125 m.y.; the Luobusa, Dongqiao, Dangqiong, Xigaze, Dongbo, Zedang, and Purang ophiolites); the Mirdita ophiolite (c.a. 160 m.y.) in Albania; the Myitkynia ophiolite (ca. 170 m.y.) in Myanmar; the Hegenshan ophiolite (ca. 295 m.y.) in inner Mongolia, China; the Sartohay ophiolite (ca. 395 m.y.), part of the West Jungar belt, Xianjiang, China; and the Ray-Iz ophiolite (ca. 420 m.y.), in polar Ural, Russia.

### Diamond-bearing ophiolites

A majority of diamond-bearing ophiolites are dismembered/mélange ophiolites^60,104–108^, with the exceptions of the Mirdita and Pozanti-Karsanti ophiolites which are often described as Penrose-type ophiolites^107,109^. We compiled the geological and geochemical information for the mafic and ultramafic rocks (when available) of these ophiolites (Fig. 3 and Table S1, Figures S3 and S4). The mafic rocks, representing the oceanic crust, and the ultramafic rocks, representing the oceanic lithospheric mantle, from all ophiolites share geochemical characteristics of deep melting products (Fig. 3). The mafic rocks are characterised by a garnet peridotitic source rock, illustrated by their high TiO2/Yb, Nb/Yb and Th/Yb (Fig. 3a and Figure S3), with major and trace element compositions similar to ocean island basalts and oceanic plateau basalts (Fig. 3a; Figures S2 and S3).

The ultramafic rocks consist of harzburgite and subordinate lherzolite and dunite. The lherzolites and dunites likely represent the products of post-melting metasomatism and melt-rock interaction, and are therefore not representative of the unaltered lithospheric mantle^110^. The harzburgites, representing the lithospheric mantle, are characterised by high Mg# ([Mg/(Mg + FeOt)at] > 0.91), low Al_2_O_3_ (1.5–0.2 wt%) and very minor SiO_2_ enrichments (Fig. 3b). Despite evidence for metasomatic enrichment in some harzburgites (e.g. enrichment of FeOt to > 9 wt%, Fig. 3b and S4), the least affected, and most refractory samples indicate an anhydrous melting origin by at least 30% of melt extraction at depths > 3 GPa (Fig. 3b)^68^. Such a deep melting origin is supported by the absence of silica enrichment^111^ (Figure S4), an indicator for SSZ peridotites (Figure S2). In addition, garnet-breakdown features (i.e., spinel symplectite texture) have been reported in ophiolites from the Yarlung Zhangbo belt^112^, indicative of deep melting.

### Diamond classification

In this study we classify diamonds based on (1) nitrogen and boron contents as well as their configuration in the diamond lattice to define the “type” classification system^113^, and (2) their inclusions^114,115^ that defines their paragenesis^3^ and ultimately their lithospheric or sub-lithospheric provenances^116,117^.

Lithospheric diamonds are diamonds formed in the continental lithospheric mantle and have mineral inclusions of eclogite and peridotite typical of continental lithosphere mantle, including forsterite, pyrope, omphacite, diopside, enstatite, and sulfides. Lithospheric diamonds are commonly subdivided into “eclogitic” and “peridotitic”, depending on the association of inclusions. For example, diamonds with almandine and omphacite inclusions are classified as eclogitic while diamonds with forsterite and pyrope are classified as peridotitic.

Superdeep (or sub-lithospheric) diamonds have mineral inclusions typical of the upper mantle, including ferro-periclase, CaSiO_3_-walstromite, jeffbenite, majoritic garnet and retrogressed bridgmanite. Superdeep diamond inclusions also indicate the depth of the diamonds, for example, diamonds with majoritic inclusions are believed to have come from the transition zone while diamonds with retro-morphosed bridgmanite are interpreted to have come from the lower mantle (a much rarer occurrence).

## Supplementary Information

Supplementary Table S1.

Supplementary Informations.

## Data Availability

Data needed to evaluate the conclusions in the paper are presented in the paper and/or the Supplementary Materials.
