# Supplementary material for: Oceanic and super-deep continental diamonds share a transition zone origin and mantle plume transportation
Source: Sci Rep. 2021 Aug 20;11:16958. doi: 10.1038/s41598-021-96286-8 (PMC8379195; doi:10.1038/s41598-021-96286-8)
Supplement: Supplementary file 2 — Supplementary Informations. [file 41598_2021_96286_MOESM2_ESM.pdf]

Supplementary Information

Title

Oceanic and super-deep continental diamonds share a transition zone origin and mantle plume transportation

Authors

Luc S. Doucet*, Zheng-Xiang Li* and Hamed Gamal El Dien

Affiliations

Earth Dynamics Research Group, TIGeR, School of Earth and Planetary Sciences, Curtin University, Perth WA 6845, Australia.

*luc-serge.doucet@curtin.edu.au, z.li@curtin.edu.au

Supplementary Materials

This **Supplementary Material** contains the geological and geochemical descriptions of the crustal and mantle sections of each diamond-bearing ophiolite, the geochemical data (Table S1), a schematic sketch showing the potential crustal and mantle components of ophiolitic belts with varying tectonic settings (Figure S1), the geochemical characteristics of mafic and ultramafic rocks from crustal and mantle sections of oceanic lithosphere (Figure S2), as well as the geochemical characteristics of mafic (Figure S3) and ultramafic rocks (Figure S4) from each diamond-bearing ophiolite.

Information Note

Geological and geochemical description of the crustal and mantle sections of each diamond-bearing ophiolite

The Yarlung-Zhanbo belt in southern Tibet marks the Eocene collision between the Indian continent and the Lhasa block of Eurasia, 100–65 million years ago (Ma). This belt contains dismembered fragments of ophiolitic blocks at Xigaze, Purang, Dangqiong, Lubuosa, Dongbo, Zedang, and Dongqiao. Recent publications[1,2] demonstrate that the ophiolite bodies in the Yarlung-Zhanbo belt are the remnants of a massive and long-lived Meso-Tethyan oceanic plateau similar to the Kerguelen plateau[3]. The ophiolite blocks contain a large volume of pillow lavas, mafic and ultramafic intrusive rocks, cumulates and radiolarian cherts. The reconstructed basaltic sequences, together with ultramafic rocks, represent a crustal thickness of more than 10 km. The ophiolite blocks contain high-MgO basalts (picrites), komatiitic rocks, and highly depleted lithospheric mantle rocks. The preserved volcanic sequences show evidence for subaerial eruptions, e.g. conglomerates, fossil soil horizons, vesicular and amygdaloidal structures, accretionary lapilli, tuffaceous horizons, and reworking in shallow water. Shallow water limestones, oolitic and bioclastic deposits in contact with plateau basalts mark the base of the sedimentary sequence of dominantly basaltic or tuffaceous breccia and volcanic sandstone. Geochronological data ($^{40}Ar/^{39}Ar$ ages on plagioclase separates and groundmass, and U-Pb zircon ages) and paleontological dating indicate three major magmatic events at 180 Ma, 165 Ma and 120 Ma. Also, the recent paper of Gong, et al. [4] shows that mantle fragments from the Purang ophiolite exhibit features of garnet-breakdown which suggests that the mantle section of the Yarlung-Zhangbo ophiolites was formed and stabilised at a depth of ~85–100 km. The mafic rocks from the Yarlung-Zhanbo belt exhibit strong OIB to oceanic plateau signatures (Figure S3a, b, c). The geochemical composition of the least altered mantle rocks is best explained by a high degree of melting (probably ≥30%) at

pressures >3 GPa, followed by later interactions with subduction fluids and melts (Figure S4a, b, c).

The Hegenshan ophiolite mélange is located in the Inner Mongolia-Daxinganling Orogenic Belt (eastern section of the Central Asian Orogenic Belt), and consists of several ophiolite bodies. Each body is made of serpentinite with lenses of serpentinised harzburgite, serpentinised dunite, podiform chromitite, cumulate gabbro, and mafic lava and dykes, intruded by granodiorite dykes[5]. Few geochemical analyses have been reported for the mafic and ultramafic rocks within the ophiolite mélange (Table S1). Geochronological data ($^{40}Ar/^{39}Ar$ data on groundmass, and U-Pb zircdon ages) indicate a magmatic age of 295–290 Ma[6]. Available data for the gabbro and mafic dykes suggest that the mafic lavas can be divided into two main groups, one made of metamorphosed basalts with flat REE patterns relative to the primitive mantle, and one made of non-metamorphosed basalts with REE patterns similar to ocean island basalts (Figure S3f). The first group exhibit typical MORB signatures, whereas the second group has geochemical characteristics of OIB (Figure S3d, e). Available data for the serpentinised harzburgites (least altered mantle samples in the ophiolite mélange) show that the mantle section is made of depleted mantle melting residue, with low Al_2O_3 (<1 wt.%), low FeOt (<7.5 wt.%), "normal" SiO_2 (~44 wt.%), and depleted heavy rare earth elements relative to the primitive mantle (Figure S4d, e, f). Also, the serpentinised harzburgites exhibit garnet-breakdown features[7] which suggests that the mantle section of the Hegenshan ophiolite was formed and stabilised at a depth of ~85–100 km[7]. The geochemical compositions of the mantle rocks are best explained by a high-degree melting (≥30%) at high pressure (>3 GPa), and the absence of silica enrichment indicates that the mantle rocks from the Hegenshan ophiolite did not experience melt-rock interaction with subduction fluid/melts[8].

The Sarthohay ophiolite mélange is located in West Jungar, Xinjiang, China. The region is characterised by the occurrence of linear zones of Paleozoic ophiolitic mélanges, representing fragments of the oceanic lithosphere emplaced during the formation of the Central Asian Orogenic Belt[9]. The Sarthohay ophiolite mélange exhibits a linear distribution along the Darbut fault, and is made of ultramafic rocks, podiform chromitites, pillow lavas, radiolarian cherts, and subordinate gabbros and plagiogranites[9]. The ultramafic rocks are highly serpentinised harzburgite (completely transformed into serpentinite or listwaenite, a carbonate-rich serpentinite) with a minor amount of serpentinised lherzolite, serpentinised dunite and lenses of serpentinised pyroxenite[9]. Pillows and massive lavas occur in extrusive sequences that tectonically overlie the peridotites. A few bands of red chert and tuffaceous sandstones interlayer with the lavas (basalt-jasper interlayers)[10]. Radiolarian cherts indicate a Devonian age which is consistent with the Sm-Nd isochron age of 395 ± 12 Ma obtained on gabbros and plagiogranites in the ophiolite[11]. The geochemical compositions of the mafic rocks from the Sarthohay ophiolite mélange suggest oceanic to continental subduction enrichments, but the most pristine samples (devoid of melt-rock interaction) exhibit deep melting and OIB to oceanic plateau signatures (Figure S3g, h, i). The mantle rocks have low Al_2O_3 (<1 wt.%) but relatively "normal" to enriched FeOt content (from 8 to >12 wt.%) which suggests an origin of 20 to 25% of partial melting followed by iron enrichment through metasomatism. However, the mantle rocks from the Sarthohay ophiolite show a strong depletion in SiO_2 (<44 wt.%, unrelated to CaO content and therefore not controlled by carbonate precipitation), indicating an anhydrous melt extraction. There is therefore no evidence for subduction fluids or melts. To our knowledge, no trace element data is available for the mantle rocks of the Sartohay ophiolite.

The Mirdita ophiolite is located in Albania (Eastern Europe), and it is a Jurassic ophiolite that represents the remnant of the Tethyan ocean[12]. The Mirdiata ophiolite occurs in a 30–40 km-wide belt bounded by the conjugate passive margin sequences of Apulia in the west and Korabi–Pelagonia in the east[13]. Large bodies of peridotite are exposed in the western and eastern parts of the ophiolite belt, consisting of harzburgite, harzburgite-dunite interlayers, and dunite with extensive chromitites. Gabbros coexist with the peridotite bodies. Isotropic gabbros and sheeted dykes are rare in the crustal section, and mylonitic peridotites and deformed gabbros are locally overlain by basaltic lavas[12]. Boninitic dykes/lavas commonly crosscut/overlie the earlier formed extrusive rocks, indicating that they are among the latest magmatic products in crustal accretion of the eastern ophiolite belt[13]. Geochronological data ($^{40}Ar/^{39}Ar$) on both the metamorphic sole and igneous plutonic rocks from the Mirdita ophiolite indicate a magmatic age of 174–162 Ma [14,15]. The metamorphic sole below the Mirdita ophiolite are made of amphibolites of OIB affinity[16]. The geochemical compositions of the mafic rocks from the Mirdita ophiolites indicate strong interaction with oceanic subduction fluids/melts, but still with the characteristics of deep-sourced OIB signature (Figure S4j, k, l). The geochemical compositions of mantle rocks show that the Mirdita peridotites are moderately depleted in Al2O3 (<1.5 wt.%) and have "normal" FeOt content (~8 wt.%), suggesting >20% of partial melting at a moderate depth (2 GPa). The absence of silica enrichment suggests no interaction with subduction fluids and melts (Figure S4 j, k, l).

The Pozanti-Karsanti ophiolite is located in the Tauride belt in southern Turkey, and represents a remnant of the Mesozoic Neotethyan ocean[17]. It consists of three distinct nappes, an ophiolite mélange, a metamorphic sole, and ophiolitic rocks[17]. The Pozanti-Karsanti ophiolitic rocks in the Aladag region are made of mantle peridotites, ultramafic and mafic cumulates, isotropic gabbros, sheeted dykes and basaltic pillow lavas[17]. The Pozanti-Karsanti ophiolite directly overlies the metamorphic sole, made of amphibolite, with an intra-oceanic OIB origin[18]. Geochronological data ($^{40}Ar/^{39}Ar$ on hornblende separates) indicate an emplacement age of 94–90 Ma[18]. Few data are available for the mafic rocks; however, these data not only indicate strong interactions with oceanic and continental subduction fluids, but also suggest deep melting with $TiO_2/Yb>0.1$ and flat REE patterns relative to the primitive mantle, i.e. similar to oceanic plateaus (S3m, n, o). The geochemical compositions of mantle peridotites from the Pozanti-Karsanti ophiolite indicate a high degree of partial melting (30%) at low to moderate pressure (3 GPa) (Figure S4m, n, o), and show evidence of interactions with subduction melts/fluids resulting in silica enrichment (Figure S4 n). However, the presence of spinel-pyroxene symplectic textures suggests that melt extraction took place in the garnet stability field at a depth higher than ~85–100 km[19].

The Myitkyina ophiolite in Myanmar is a dismembered Jurassic (rock age) ophiolite[20]. The Myitkyina ophiolite is made of several massifs of mantle peridotites with rare red radiolarian cherts and pillow basalts[21]. Mafic plutonic rocks (e.g., gabbro) are not found anywhere in the ophiolite. The dismembered nature of the Myitkyina ophiolite makes it difficult to ascertain the original stratigraphy and structures. Mantle peridotites of the Myitkyina ophiolite consist mainly of harzburgites with minor lherzolites/dunites, and exhibit rare coarse-grained gabbro and plagiogranite intrusions with a maximum width of up to 5cm. Geochronological data (U-Pb zircon) on igneous plutonic fragments indicate a magmatic age of 176–166 Ma[21]. Late Jurassic (i.e., middle-upper Tithonian) ages were estimated from radiolarian cherts[22]. The Myitkyina ophiolite has been regarded as the

eastern continuation of the Yarlung-Zhanbo belt[23], and is therefore probably of the same oceanic plateau origin. The few geochemical compositions available for the mafic rocks indicate interactions with subduction fluids/melts, but still with characteristics of a deep-sourced oceanic plateau with TiO_2/Yb>0.1 and flat REE patterns relative to the primitive mantle (Figure S4p, q, r). The geochemical compositions of mantle rocks from the Myitkyina ophiolite indicate a range of degrees of partial melting (10–30%) at low pressure (1–2 GPa), and are characterised by the absence of silica enrichments (Figure S4p, q, r).

The Rai-Iz ophiolite mélange is part of the Paleozoic Voikar–Syninsk ophiolite belt in the Polar Ural in Russia. The Ray-Iz massif is a funnel-shaped body, with a northeast margin made of a thick (500 m) mélange containing blueschist and jadeite[24]. To the south, the massif is overthrust by a basement complex composed of dunite, websterite, pyroxenite, metagabbro and amphibolite [24]. Sheeted dykes and pillow lavas are absent from the Ray-Iz massif[25]. The Ray-Iz ophiolite is mainly made of lherzolite-harzburgite, with about 10% of dunite[26]. Geochronology (whole-rock/mineral Sm-Nd isochrones and Re-Os depletion ages) yield a range of formation ages of 470–410 Ma[27,28]. To our knowledge, no geochemical data are available for the mafic rocks. The geochemical composition of the mantle rocks suggests a high degree of partial melting (30–25%) at a moderate to high-pressure (2-4 GPa), with no direct evidence for interaction with subduction melts/fluids (Figure S4s, t, u).

Table S1: Major and trace element composition of mafic and ultramafic rocks of diamond-bearing ophiolites.

Figure S1: Schematic sketch showing the potential crustal and mantle component of ophiolitic belts with varying tectonic settings. (1) old continental arc, (2) back-arc basin, (3) older oceanic arc, (4) oceanic arc, (5) oceanic plateau, (6) ocean islands and seamounts, (7) normal oceanic crust, (8) mid-ocean ridge. Also shown are the characteristics of the crust and mantle lithosphere of oceanic plateau.

Figure S2: Geochemical characteristics of mafic and ultramafic rocks from crustal and mantle sections of oceanic lithosphere. Mafic rocks with Nb/Yb vs. Th/Yb (a), Nb/Yb vs. TiO_2/Yb (b), and primitive mantle normalised rare earth elements patterns (c). Mantle rocks with Al_2O_3 vs FeOt (d), Al_2O_3 vs. SiO_2 (e), and primitive mantle normalised rare earth element pattern (f).
The data for oceanic arc, ocean island basalts (OIB), oceanic flood basalts (OFB) and mid-ocean ridge basalts (MORB) were sourced from the online Earthchem portal (http://www.earthchem.org/portal) and GEOROC (http://georoc.mpchmainz.gwdg.de/georoc). The data for suprasubduction peridotites (SSZ), abyssal peridotites and ocean island peridotites are from the compilation of Doucet, et al.[29]. The data for oceanic plateau peridotites are from Simon et al. [30] and Wasilewski et al.[31].

Figure S3: Geochemical characteristics of mafic rocks from each diamond-bearing ophiolite. Nb/Yb vs. Th/Yb (left column), Nb/Yb vs. TiO_2/Yb (center) and primitive mantle normalised rare earth elements patterns (right column). Also shown are the oceanic basalt discriminant fields of Pearce [32] (left and center columns).

Fig. S4: Geochemical characteristics of mantle rocks from each diamond-bearing

ophiolite. Al_2O_3 vs. FeOt (left column), Al_2O_3 vs. SiO_2 (center) and primitive mantle normalised rare earth elements patterns (right column). Also shown are the experimental melting residue for polybaric fractional melting of fertile mantle (left and center columns)[33].

1 Zhang, K.-J. *et al.* Central Tibetan Meso-Tethyan oceanic plateau. *Lithos* **210-211**, 278-288, doi:https://doi.org/10.1016/j.lithos.2014.09.004 (2014).

2 Yang, G. & Dilek, Y. OIB-and P-type ophiolites along the Yarlung-Zangbo Suture Zone (YZSZ), Southern Tibet: Poly-Phase melt history and mantle sources of the Neotethyan oceanic lithosphere. *Episodes* **38**, 250-265 (2015).

3 Mattielli, N. *et al.* Kerguelen basic and ultrabasic xenoliths: Evidence for long-lived Kerguelen hotspot activity. *Lithos* **37**, 261-280 (1996).

4 Gong, X. H. *et al.* "Garnet" Lherzolites in the Purang Ophiolite, Tibet: Evidence for Exhumation of Deep Oceanic Lithospheric Mantle. *Geophysical Research Letters* **47**, e2019GL086101 (2020).

5 Nozaka, T. & Liu, Y. Petrology of the Hegenshan ophiolite and its implication for the tectonic evolution of northern China. *Earth and Planetary Science Letters* **202**, 89-104, doi:https://doi.org/10.1016/S0012-821X(02)00774-4 (2002).

6 Miao, L. *et al.* Geochronology and geochemistry of the Hegenshan ophiolitic complex: Implications for late-stage tectonic evolution of the Inner Mongolia-Daxinganling Orogenic Belt, China. *Journal of Asian Earth Sciences* **32**, 348-370, doi:https://doi.org/10.1016/j.jseaes.2007.11.005 (2008).

7 Jiang, J. & Zhu, Y. Harzburgite found in the Hegenshan ophiolite, southeastern Central Asian Orogenic Belt: Petrogenesis and geological implications. *Gondwana Research* (2019).

8 Bénard, A., Arculus, R. J., Nebel, O., Ionov, D. A. & McAlpine, S. R. B. Silica-enriched mantle sources of subalkaline picrite-boninite-andesite island arc magmas. *Geochim. Cosmochim. Acta* **199**, 287-303, doi:https://doi.org/10.1016/j.gca.2016.09.030 (2017).

9 Peng, G. Podiform chromite and associated ophiolitic rocks in West Junggar, Xinjiang, China. *PhD thesis* (1995).

10 Zhou, M.-F. *et al.* Melt/mantle interaction and melt evolution in the Sartohay high-Al chromite deposits of the Dalabute ophiolite (NW China). *Journal of Asian Earth Sciences* **19**, 517-534 (2001).

11 Wei, W., Dong, X., Zeng, H. & Gao, J. The geological characters and genesis of ultrabasic rock mass and chromite deposit in Sartohay of Xinjiang, China. *Bulletin of Xi'an Institute of Geology, Chinese Academy of Geological Sciences* **16**, 57-145 (1987).

12 Dilek, Y., Furnes, H. & Shallo, M. Geochemistry of the Jurassic Mirdita Ophiolite (Albania) and the MORB to SSZ evolution of a marginal basin oceanic crust. *Lithos* **100**, 174-209 (2008).

13 Dilek, Y., Shallo, M. & Furnes, H. Rift-drift, seafloor spreading, and subduction tectonics of Albanian ophiolites. *International Geology Review* **47**, 147-176 (2005).

14 Vergely, P., Dimo, A. & Monié, P. Datation des semelles metamorphiques ophiolitiques d'Albanie par la methode 40Ar/39Ar: Consequences sur le mecanisme de leur mise en place. *Comptes Rendus de l'Académie des Sciences-Series IIA-Earth and Planetary Science* **326**, 717-722 (1998).

15 Dimo-Lahitte, A., Monié, P. & Vergély, P. Metamorphic soles from the Albanian ophiolites: Petrology, 40Ar/39Ar geochronology, and geodynamic evolution. *Tectonics* **20**, 78-96 (2001).

16 Gaggero, L., Marroni, M., Pandolfi, L. & Buzzi, L. Modelling of oceanic lithosphere obduction: constraints from the metamorphic sole of Mirdita ophiolites (Northern Albania). *Ofioliti* **34**, 17-42 (2009).
17 Parlak, O., HÖck, V. & Delaloye, M. Suprasubduction zone origin of the Pozanti-Karsanti ophiolite (southern Turkey) deduced from whole-rock and mineral chemistry of the gabbroic cumulates. *Geological Society, London, Special Publications* **173**, 219-234 (2000).
18 Celik, O. F., Delaloye, M. & Feraud, G. Precise 40Ar–39Ar ages from the metamorphic sole rocks of the Tauride Belt Ophiolites, southern Turkey: implications for the rapid cooling history. *Geological Magazine* **143**, 213-227 (2006).
19 Saka, S., Uysal, I., Akmaz, R. M., Kaliwoda, M. & Hochleitner, R. The effects of partial melting, melt–mantle interaction and fractionation on ophiolite generation: Constraints from the late Cretaceous Pozantı-Karsantı ophiolite, southern Turkey. *Lithos* **202**, 300-316 (2014).
20 Mitchell, A. Cretaceous–Cenozoic tectonic events in the western Myanmar (Burma)–Assam region. *Journal of the Geological Society* **150**, 1089-1102 (1993).
21 Xu, Y. *et al.* Petrogenesis and tectonic implications of gabbro and plagiogranite intrusions in mantle peridotites of the Myitkyina ophiolite, Myanmar. *Lithos* **284-285**, 180-193, doi:https://doi.org/10.1016/j.lithos.2017.04.014 (2017).
22 Maung, M., Aung, N. T. & Suzuki, H. in *Regional Congress on Mineral and Energy Resources of Southeast Asia. GEOSEA.* 38-39.
23 Yang, J. *et al.* Discovery of a Jurassic SSZ ophiolite in the Myitkyina region of Myanmar. *Acta Petrologica Sinica* **28**, 1710-1730 (2012).
24 Kazak, A., Dobretsov, N. & Moldavantsev, Y. E. Glaucophane schists, jadeitites, vesuvianites and nephrites of the ultrabasitic Rai-Iz massif. *Geology and Geophysics* **2**, 60-66 (1976).
25 Garuti, G., Zaccarini, F., Moloshag, V. & Alimov, V. Platinum-group minerals as indicators of sulfur fugacity in ophiolitic upper mantle; an example from chromitites of the Ray-Iz ultramafic complex, Polar Urals, Russia. *The Canadian Mineralogist* **37**, 1099-1115 (1999).
26 Makeyev, A., Perevozchikov, B. & Afanacyev, A. (1985).
27 Walker, R. J., Prichard, H. M., Ishiwatari, A. & Pimentel, M. The osmium isotopic composition of convecting upper mantle deduced from ophiolite chromites. *Geochim. Cosmochim. Acta* **66**, 329-345 (2002).
28 Ronkin, Y. L., Priamonocov, A., Telegina, T. & Lepehina, O. in *Isotopic Dating of Geological Process: New Methods and Results. Abstract of Report I Russian Conference on Isotopic Geochronology.* 302-305.
29 Doucet, L. S. *et al.* Coupled supercontinent–mantle plume events evidenced by oceanic plume record. *Geology* **48.2**, 159-163 (2020).
30 Simon, N. S. C. *et al.* Ultra-refractory Domains in the Oceanic Mantle Lithosphere Sampled as Mantle Xenoliths at Ocean Islands. *J. Petrol.* **49**, 1223-1251 (2008).
31 Wasilewski, B. *et al.* Ultra-refractory mantle within oceanic plateau: Petrology of the spinel harzburgites from Lac Michèle, Kerguelen Archipelago. *Lithos* **272-273**, 336-349, doi:https://doi.org/10.1016/j.lithos.2016.12.010 (2017).
32 Pearce, J. A. Geochemical fingerprinting of oceanic basalts with applications to ophiolite classification and the search for Archean oceanic crust. *Lithos* **100**, 14-48, doi:https://doi.org/10.1016/j.lithos.2007.06.016 (2008).
33 Herzberg, C. Geodynamic information in peridotite petrology. *J Petrol* **45**, 2507-2530 (2004).

Supplementary Figure S1:
Schematic sketch showing the potential crustal and mantle components of ophiolitic belts with varying tectonic settings

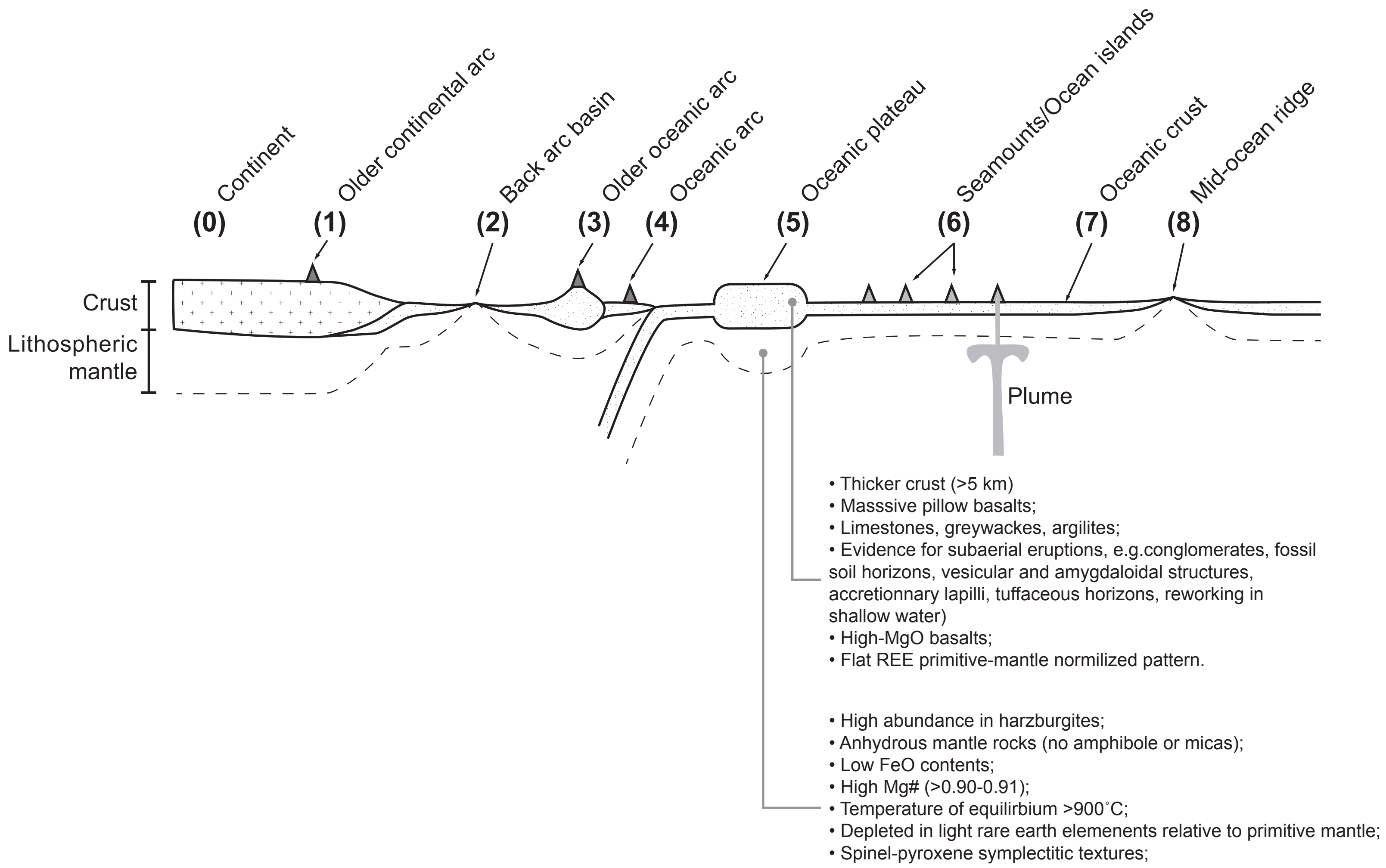

Supplementary Figure S1: Schematic sketch showing the potential crustal and mantle components of ophiolitic belts with varying tectonic settings. (1) old continental arc, (2) back-arc basin, (3) older oceanic arc, (4) oceanic arc, (5) oceanic plateau, (6) ocean islands and seamounts, (7) normal oceanic crust, (8) mid-ocean ridge. Also shown are the characteristics of the crust and mantle lithosphere of oceanic plateau.

Supplementary Figure S2: Geochemical characteristics of mafic and ultramafic rocks from crustal and mantle sections of oceanic lithosphere

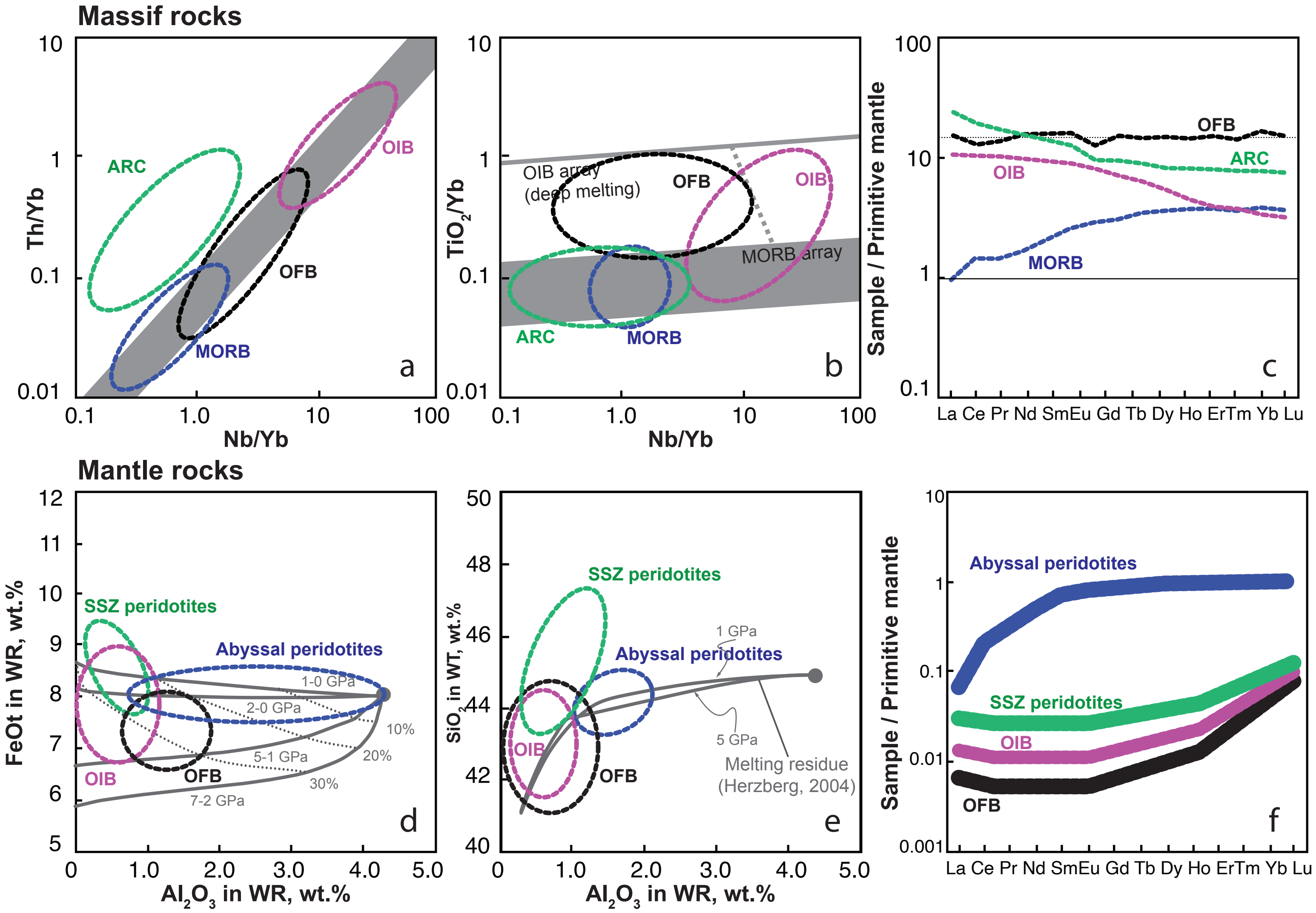

Supplementary Figure S2: Geochemical characteristics of mafic and ultramafic rocks from crustal and mantle sections of oceanic lithosphere. Mafic rocks with Nb/Yb vs. Th/Yb (a), Nb/Yb vs. TiO_2/Yb (b), and primitive-mantle normalized rare earth elements patterns (c). Mantle rocks with Al_2O3 vs FeOt (d), Al_2O_3 vs. SiO_2 (e), and primitive-mantle normalized rare earth element pattern (f). The data for oceanic arc, ocean island basalts (OIB), oceanic flood basalts (OFB) and mid-ocean ridge basalts (MORB) were sourced from the online Earthchem portal (http://www.earthchem.org/portal) and GEOROC (http://georoc.mpch-mainz.gwdg.de/georoc). The data for suprasubduction peridotites (SSZ), abyssal peridotites and ocean island peridotites are from the compilation of Doucet, et al.[65]. The data for oceanic plateau peridotites are from Simon, et al.[51] and Wasilewski, et al.[66].

Supplementary Figure S3:
Geochemical characteristics of mafic rocks from each diamond-bearing ophiolite

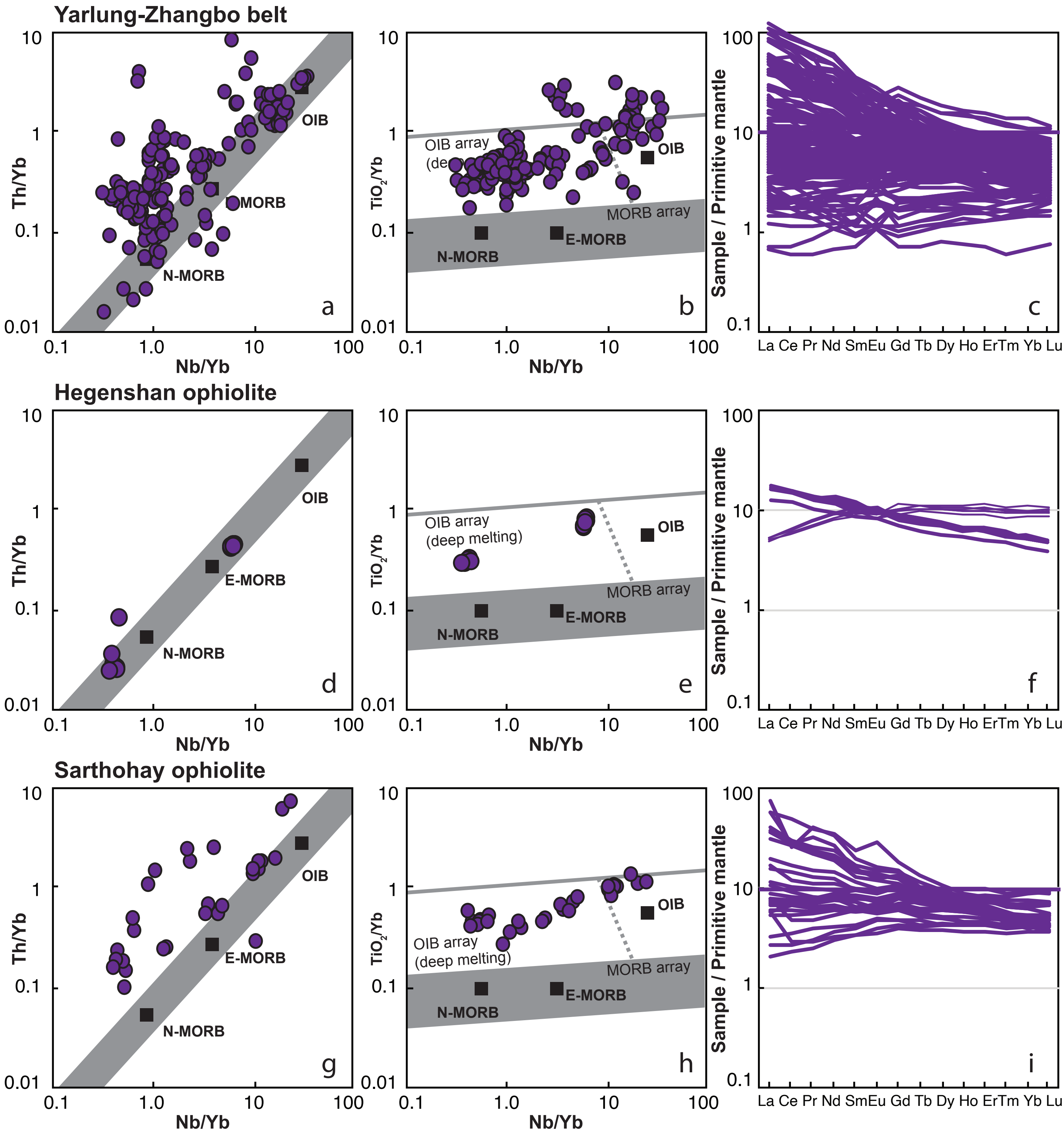

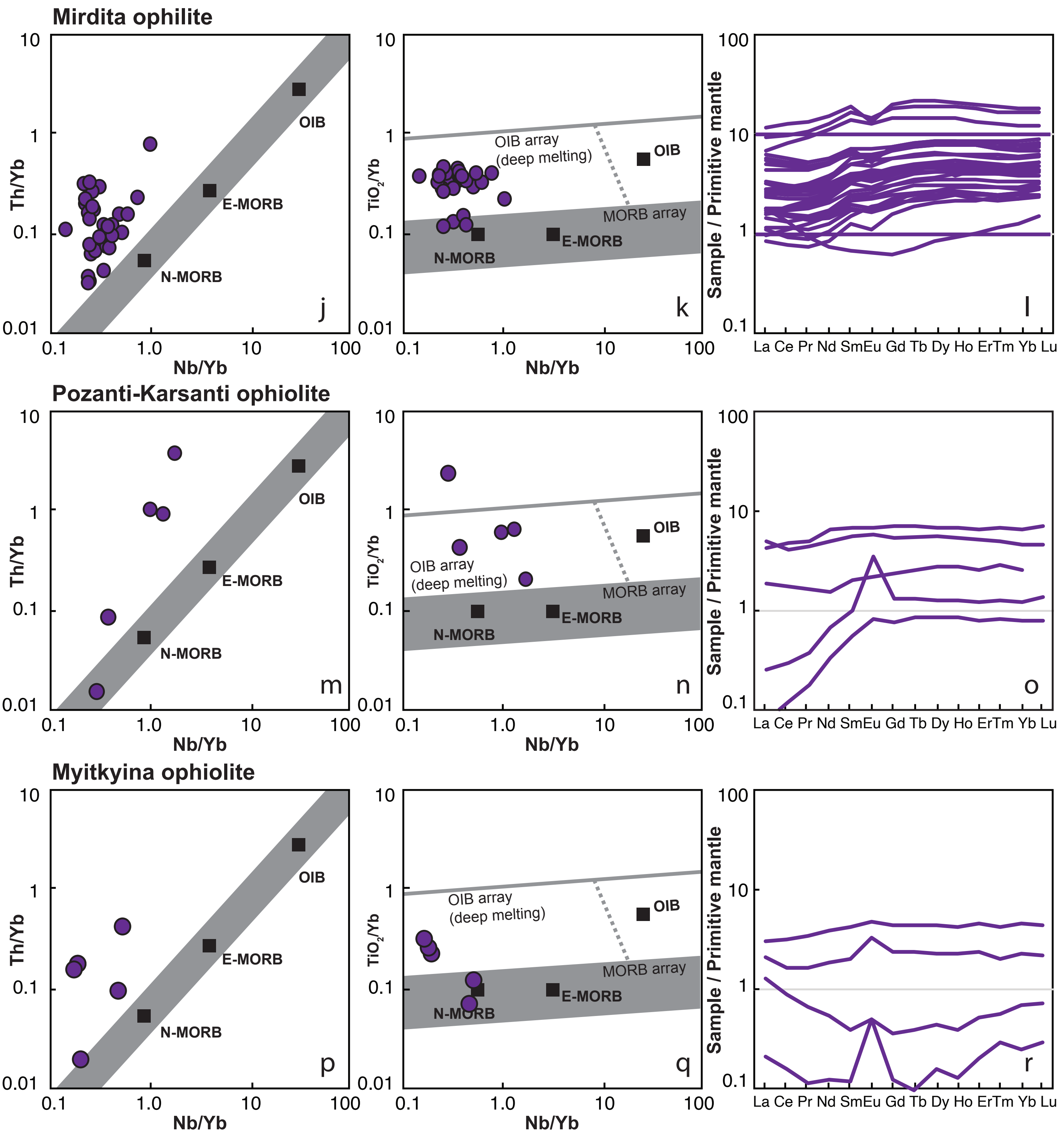

Supplementary Figure S3: Geochemical characteristics of mafic rocks from each diamond-bearing ophiolite. Nb/Yb vs. Th/Yb (left column), Nb/Yb vs. TiO2/Yb (center) and primitive-mantle normalized rare earth elements patterns (right column). Also shown are the oceanic basalt discriminant fields of Pearce[63] (left and center columns).

Supplementary Figure S4: Geochemical characteristics of mantle rocks from each diamond-bearing ophiolite

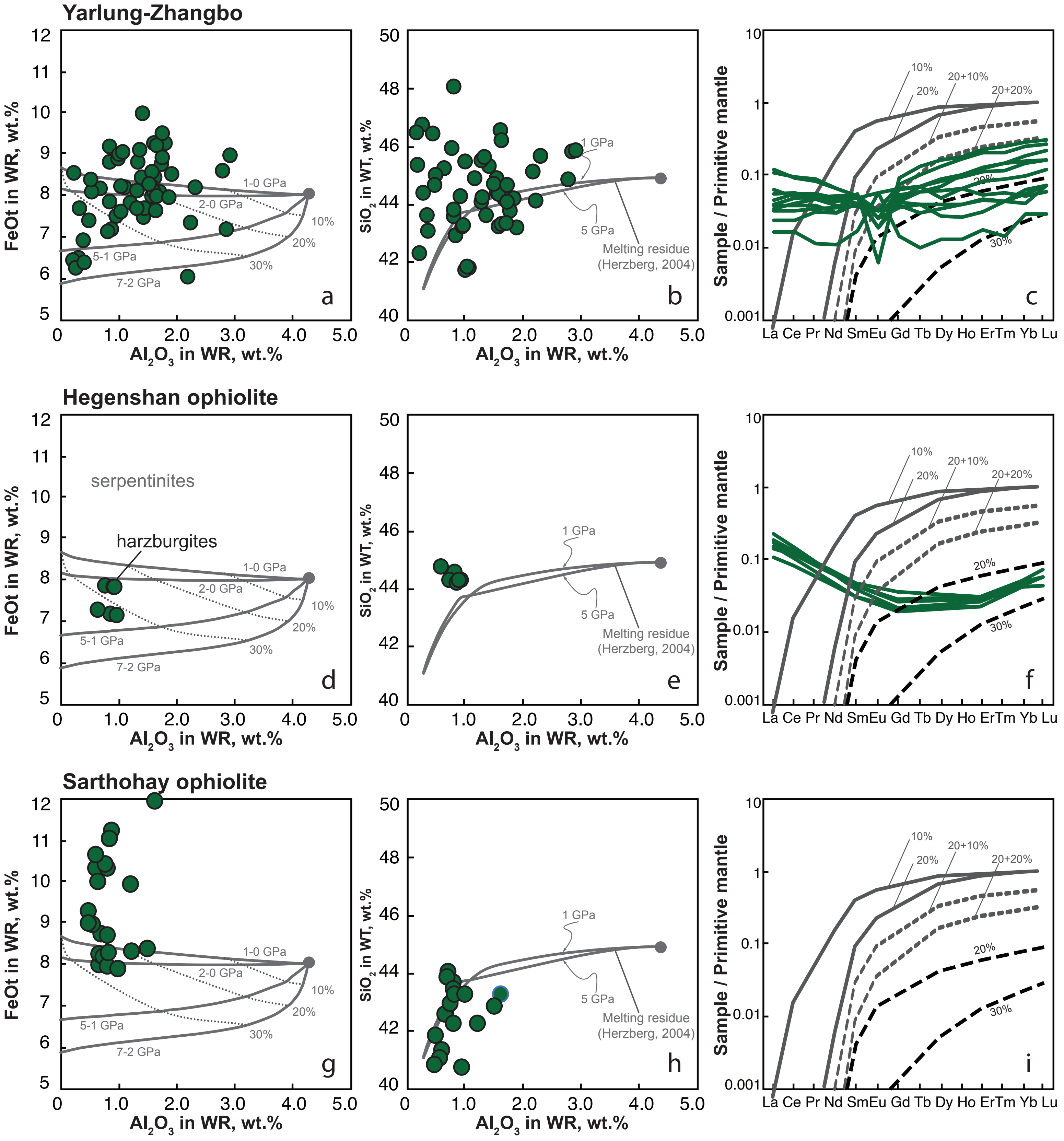

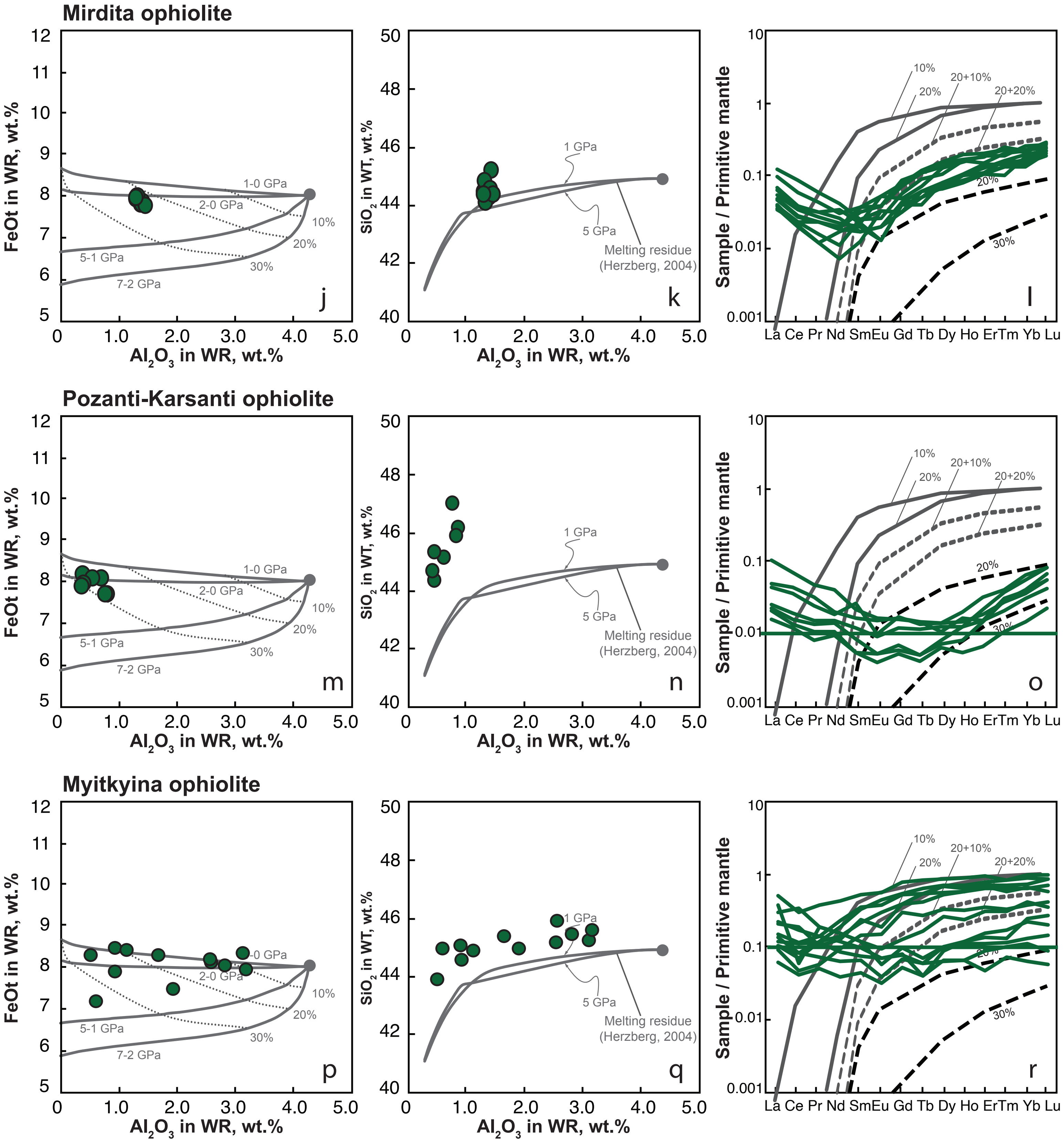

Mirdita ophiolite
FeOt in WR, wt.%
Al2O3 in WR, wt.%
1-0 GPa
2-0 GPa
5-1 GPa
7-2 GPa
10%
20%
30%
j
SiO2 in WT, wt.%
1 GPa
5 GPa
Melting residue
(Herzberg, 2004)
k
Sample / Primitive mantle
10%
20%
20+10%
20+20%
30%
La Ce Pr Nd SmEu Gd Tb Dy Ho ErTm Yb Lu
l
Pozanti-Karsanti ophiolite
m
n
o
Myitkyina ophiolite
p
q
r

Ray-Iz ophiolite

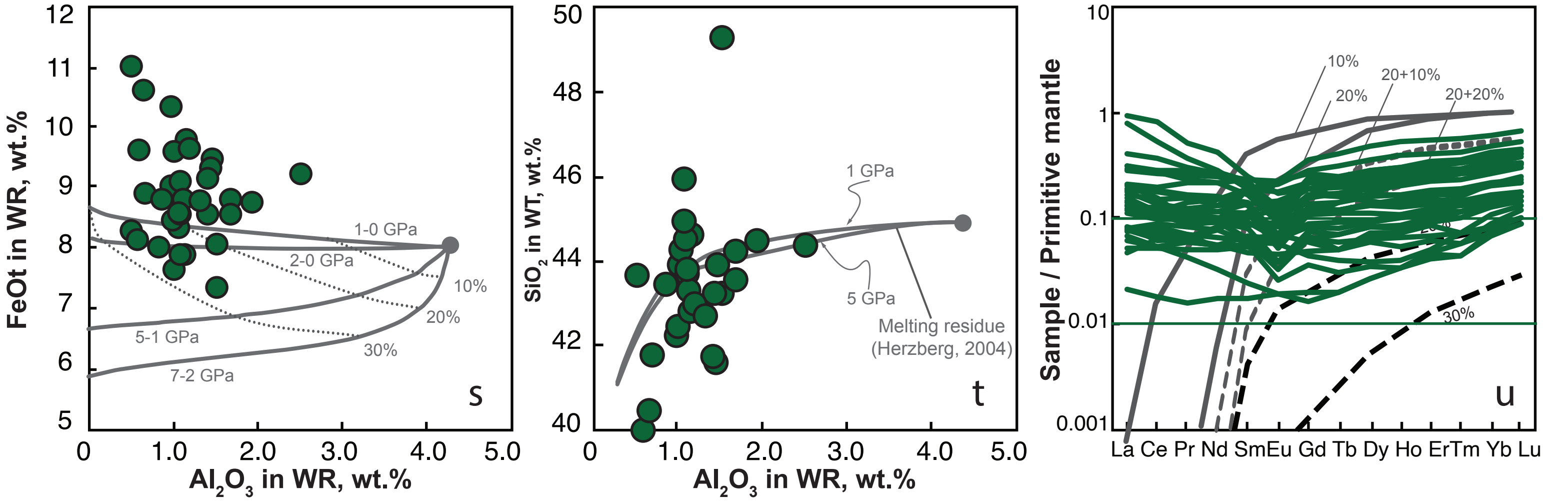

Supplementary S4: Geochemical characteristics of mantle rocks from each diamond-bearing ophiolite. Al_2O_3 vs. FeOt (left column), Al_2O_3 vs. SiO_2 (center) and primitive-mantle normalized rare earth elements patterns (right column). Also shown are the experimental melting residue for polybaric fractional melting of fertile mantle (left and center columns)[29].
